# Corto and DSP1 interact and bind to a maintenance element of the *Scr Hox *gene: understanding the role of *Enhancers of trithorax and Polycomb*

**DOI:** 10.1186/1741-7007-4-9

**Published:** 2006-04-14

**Authors:** Juliette Salvaing, Martine Decoville, Emmanuèle Mouchel-Vielh, Marianne Bussière, Anne Daulny, Lidiya Boldyreva, Igor Zhimulev, Daniel Locker, Frédérique Peronnet

**Affiliations:** 1 UMR 7622, CNRS, Université Pierre et Marie Curie, 9, quai Saint-Bernard, 75252 PARIS cedex 05, France; 2 University Maastricht, PO Box 616, 6200 MD Maastricht, The Netherlands; 3 UPR 4301, CNRS, Centre de Biophysique Moléculaire Rue Charles Sadron, 45071 Orléans cedex 2, France; 4 Cold Spring Harbor Laboratory, 1 Bungtown Road, Mc Clintock Building, 11723 NY, USA; 5 Institute of Cytology and Genetics, Siberian Division of Russian Academy of Sciences, Acad. Lavrentiev Avenue, 10, 630090 Novosibirsk, Russia

## Abstract

**Background:**

*Polycomb-group genes *(*PcG*) encode proteins that maintain homeotic (*Hox*) gene repression throughout development. Conversely, *trithorax-group *(*trxG*) genes encode positive factors required for maintenance of long term *Hox *gene activation. Both kinds of factors bind chromatin regions called maintenance elements (ME). Our previous work has shown that *corto*, which codes for a chromodomain protein, and *dsp1*, which codes for an HMGB protein, belong to a class of genes called the *Enhancers of trithorax and Polycomb *(*ETP*) that interact with both *PcG *and *trxG*. Moreover, *dsp1 *interacts with the *Hox *gene *Scr*, the DSP1 protein is present on a *Scr *ME in S2 cells but not in embryos. To understand better the role of *ETP*, we addressed genetic and molecular interactions between *corto *and *dsp1*.

**Results:**

We show that Corto and DSP1 proteins co-localize at 91 sites on polytene chromosomes and co-immunoprecipitate in embryos. They interact directly through the DSP1 HMG-boxes and the amino-part of Corto, which contains a chromodomain. In order to search for a common target, we performed a genetic interaction analysis. We observed that *corto *mutants suppressed *dsp1^1 ^*sex comb phenotypes and enhanced *Antp^Scx ^*phenotypes, suggesting that *corto *and *dsp1 *are simultaneously involved in the regulation of *Scr*. Using chromatin immunoprecipitation of the *Scr *ME, we found that Corto was present on this ME both in *Drosophila *S2 cells and in embryos, whereas DSP1 was present only in S2 cells.

**Conclusion:**

Our results reveal that the proteins Corto and DSP1 are differently recruited to a *Scr *ME depending on whether the ME is active, as seen in S2 cells, or inactive, as in most embryonic cells. The presence of a given combination of ETPs on an ME would control the recruitment of either PcG or TrxG complexes, propagating the silenced or active state.

## Background

Many transcription factors are expressed only transiently during development. After they have disappeared, the patterns of gene expression they have induced must be inherited by daughter cells. In eukaryotes, two groups of proteins, the Polycomb-group (PcG) and the Trithorax-group (TrxG), fulfil this memory function. Their existence was first revealed in *Drosophila melanogaster *where homeotic gene (*Hox*) expression is established in early embryos by the transient Gap and Pair-Rule transcription factors and controlled by PcG and TrxG proteins during the rest of development [1-3]. The PcG and TrxG proteins combine into several heteromeric complexes that bind chromatin. PcG complexes maintain *Hox *gene silencing whereas TrxG complexes counteract the action of PcG complexes (reviewed in [4]). These proteins regulate many other genes such as *engrailed *[5,6], *ph *[7], *fork head *[8] and the *iroquois*-complex [9].

In *Drosophila*, at least two PcG complexes called PRC2 and PRC1 (Polycomb Repressive Complex) and two TrxG complexes (TAC 1 and BRM) have been biochemically purified (reviewed in [10]). The PRC1 complex contains the PcG proteins Polycomb (PC), Polyhomeotic (PH), Posterior Sex Combs (PSC) and dRING1, and additional polypeptides such as the DNA binding protein Zeste [11,12]. The PRC2 complex contains the PcG proteins Extra Sex Combs (ESC), SU(Z)12 and Enhancer of Zeste (E(Z)), a histone methyltransferase that methylates lysine 27 of histone H3 (H3K27me3), and the histone binding protein p55 [13,14]. PC is also found in another complex that contains the DNA binding protein Pipsqueak [15], ESC and E(Z) are found in a larger complex that contains Polycomb-like (PCL), the histone deacetylase RPD3 and p55 [16], and E(Z) is found in a complex that contains the deacetylase SIR2 [17]. The TrxG complex TAC1 contains CBP, a member of the CBP/p300 histone acetyl tranferase (HAT) family, the anti-phosphatase SBF1, and the TrxG protein TRX, which is homologous to mammalian MLL/ALL and methylates histone H3 on lysine 4 (H3K4me) [18-20]. Lastly, the BRM complex contains the TrxG proteins BRM, Osa and Moira. It is related to the yeast SWI/SNF ATP-dependent chromatin remodeling complex, sharing four polypeptides with it, among which is the ATPase BRM [21]. It also contains BAP111, an HMGB protein that binds nonspecifically in the minor groove of the double-helix, thus bending the DNA [22,23].

In *Drosophila*, the PcG and TrxG proteins bind overlapping sequences called Polycomb/Trithorax Response Elements (PRE/TREs) (reviewed in [4]). Two PRE/TREs (*Fab-*7 and the *Hedgehog *PRE/TRE) have been demonstrated to be true maintenance elements (ME), *i. e. *to control the maintenance of activation or repression of target *loci *through cell division [24,25]. A major issue is to understand how PcG and TrxG complexes are specifically recruited to ME. On the one hand, the specific recruitment of these complexes could be achieved by recognition of posttranslationally modified histone tails. For example, PC recognizes K27 methylation of H3 *via *its chromodomain. Hence, PRC1 may be recruited to chromatin by the recognition of the H3K27me3 mark laid down by PRC2 through E(Z) [26]. Furthermore, the methyl-transferase activity of E(Z) is required for correct repression of *Hox *genes, suggesting that this histone modification is actually related to gene regulation [14]. On the other hand, DNA binding proteins such as Zeste, GAF (GAGA factor), PHO (Pleiohomeotic), the HMG-box protein DSP1 (Dorsal Switch Protein 1) and Pipsqueak, which are present at some ME, also seem to be involved in the specific recruitment of PcG and TrxG [15,27-31]. Indeed, their various combinations and associations with co-factors could make complex binding more specific.

In a large screen to identify modifiers of *trxG *mutations, 6 genes previously identified as *PcG *genes (*Asx, E(z), E(Pc), Psc, Scm *and *Su(z)2*) were isolated as enhancers of *trxG *phenotypes [32]. The authors suggested that the corresponding genes be renamed "*Enhancers of trithorax and Polycomb" *(*ETP*) to account for their role both in repression and activation of *Hox *genes. Further studies identified other ETP proteins, among which is GAF [29,33], or PHO, which directly binds a PC-containing complex as well as the BRM complex [34]. The chromodomain protein Corto [35,36] and the HMGB protein DSP1 [37] also behave as ETP. Indeed, a loss-of-function allele of *corto *enhances the macrochaete phenotype of the *trxG *gene *osa *as well as the *Polycomb *phenotype of the *PcG *genes *mxc, Pc, Pcl, ph *and the *ETP *genes *E(z) *and *Scm *[35,38]. On the other hand, a *dsp1 *null allele enhances the haltere to wing transformation of several *trxG *mutants *ash1, brm, osa, trx*), whereas male hemizygotes for this allele exhibit transformation of the A4 segment into a more posterior one, which is a *Polycomb *phenotype [37]. Thus, *dsp1 *also behaves like an *ETP*. To date, little information regarding the mode of action of ETP has become available. No ETP has yet been found in TrxG complexes. Some of them belong to PcG complexes (such as E(Z), PSC and SCM) but most of them (such as ASX, Corto, GAF and SU(Z)2) have not been found in PcG complexes to date. However, in embryonic extracts, Corto co-immunoprecipitates with ESC and PC, while SU(Z)2 and GAF co-immunoprecipitate with PC, suggesting that these ETPs can transiently interact with PcG complexes [29,36,39]. How ETPs promote either the activation or the repression of a defined target gene is an open question.

To understand better the role of ETP, we analyzed the interaction between Corto and DSP1. We show that both proteins co-localize at 91 sites on *Oregon-R *polytene chromosomes. These include 84B, which corresponds to the distal part of the *ANTP*-complex. In addition, Corto and DSP1 co-immunoprecipitate in embryonic extracts and directly interact through the HMG-boxes of DSP1. Moreover, we show that the *corto *and *dsp1 *genes interact and that *corto*, like *dsp1*, is directly involved in the regulation of the *Hox *gene *Scr*. Chromatin immunoprecipitation experiments indicate that Corto, like DSP1, localizes on a 10 kb-*Xba*I ME of the *Scr cis*-regulatory sequences. On the basis of our results, we propose that both proteins interact on chromatin to regulate a subset of common targets including *Scr*.

## Results

### DSP1 and Corto co-localize on polytene chromosomes

**Figure 1:**
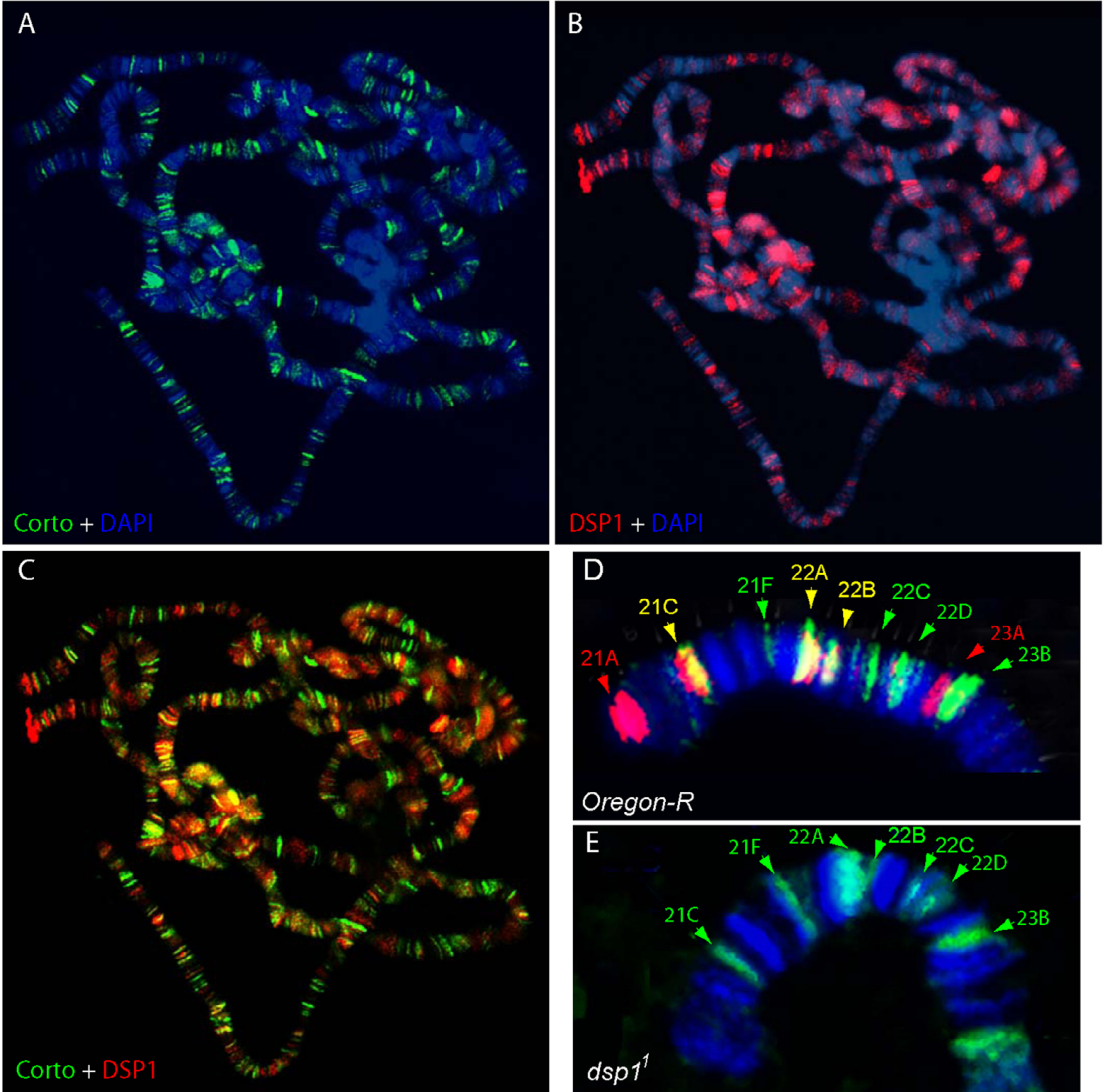
**Immunofluorescence detection of Corto and DSP1 on polytene chromosomes**. (A-C) Simultaneous detection of Corto (green) and DSP1 (red) on *Oregon-R *polytene chromosomes stained with DAPI (blue). (D, E) Magnification of chromosome 2L end of *Oregon-R *(D) or *dsp1^1 ^*(E) labeled with anti-Corto (green) and anti-DSP1 (red). Sites shared by DSP1 and Corto in *Oregon-R *are yellow.

**Figure 2:**
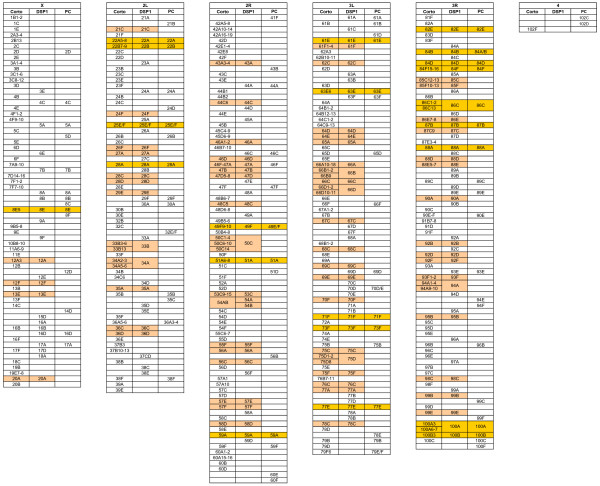
Comparison of Corto, DSP1 and PC localizations on polytene chromosomes of *Oregon-R *wild-type strain.

To address the genomic targets shared by Corto and DSP1, we first examined their binding on polytene chromosomes. Corto was previously shown to bind polytene chromosomes at many discrete euchromatic sites and to share these binding sites partially with PcG proteins such as PC and PH, or with ETPs such as E(Z), SCM and GAF [36]. Similarly, DSP1 binds polytene chromosomes at multiple loci and partially co-localizes with PH [31]. Simultaneous detection of Corto and DSP1 on chromosomes enabled us to reveal many overlapping sites (Figure 1A–C). Precise localization of Corto and DSP1 on the polytene chromosomes of *Oregon-R *flies revealed 270 euchromatic sites of antibody staining for Corto and 173 for DSP1. These sites are listed in Figure 2 and compared with the PC sites previously determined using the same strain and staining conditions [40]. On *Oregon-R *polytene chromosomes, 91 sites are shared by Corto and DSP1. Moreover, PC shares 40 sites with Corto and 42 sites with DSP1. Lastly, 24 sites are simultaneously occupied by the three proteins, notably 84B, which corresponds to the distal part of the *ANTP*-complex.

To examine whether DSP1 plays a role in Corto recruitment, we looked at the localization of Corto in the *dsp1^1 ^*homozygote mutant strain, which is devoid of DSP1 protein [37]. No modification of Corto binding sites was observed in this strain, indicating that the DNA-binding protein DSP1 is dispensable for Corto recruitment to chromatin (Figure 1D–E).

### DSP1 and Corto are parts of a common complex in embryos and interact directly

**Figure 3:**
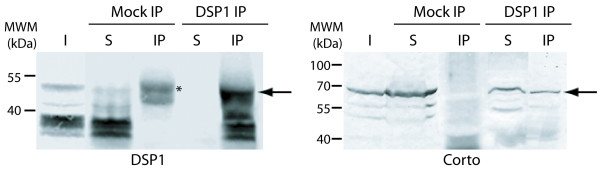
**Corto and DSP1 interact *in vivo***. Co-immunoprecipitation of Corto with DSP1. Protein extracts from 0–14 hour-old *w^1118 ^*embryos were incubated either with rabbit serum (mock IP) or with anti-DSP1 antibodies (DSP1 IP). Western blotting was performed using rabbit anti-DSP1 antibodies (left) or rat anti-Corto antibodies (right). The arrows indicate the full-length DSP1 protein (50 kDa) and Corto (68 kDa), respectively. I : Input ; S : supernatant; IP : immunoprecipitated material; *: rabbit IgG. Note that full-length DSP1 and degradation products were retained on the protein A-agarose/anti-DSP1 beads.

We also investigated whether Corto and DSP1 belong to a same molecular complex *in vivo *by performing co-immunoprecipitation assays using extracts from 0–14 hour-old embryos. As shown in Figure 3, Corto co-immunoprecipitated with DSP1, indicating that DSP1 and Corto are physically associated in embryos.

**Figure 4:**
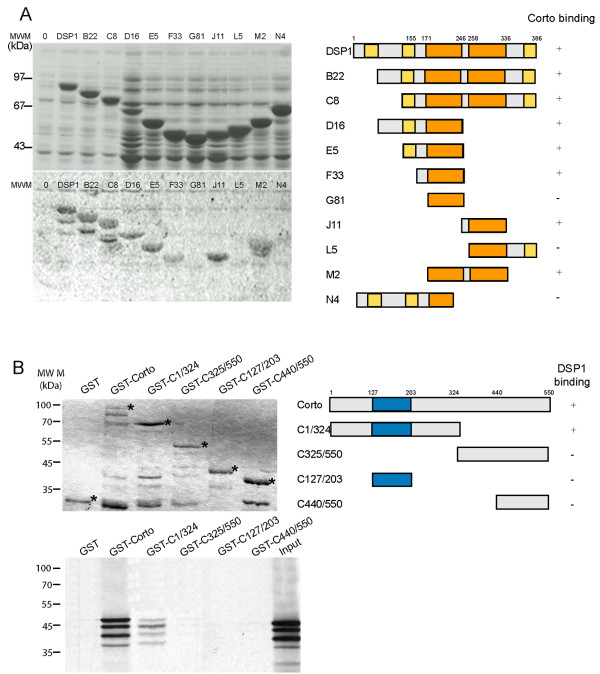
**Corto and DSP1 interact *in vitro***. (A) Far-western assays. Left, top: Coomassie-stained SDS-PAGE of the MBP-DSP1 fusion proteins. Left, bottom: Phosphorimager scan of the membrane after transfer of the proteins and incubation with radiolabeled Corto. Right: Schematic representation of DSP1 and DSP1 truncated forms (orange: HMG-A and HMG-B boxes; yellow: polyglutamine series and acidic tail). Corto is retained on MBP-DSP1 and on B22, C8, D16, E5, F33, J11 and M2 MBP-DSP1 truncated forms but not on G81, L5 and N4 MBP-DSP1 truncated forms. (B) GST pull-down assays Left, top: Coomassie blue staining of GST and GST-Corto fusion proteins (labeled with asteriks). Left, bottom: Autoradiography. ^35^S-labeled DSP1 was retained on GST-Corto and GST-C1/324 proteins and not on GST-C325/550, GST-C127/203 or GST-C440/550. Input: 1/5 of the total radioactivity was loaded. Note that the full-length DSP1 protein as well as the truncated forms (degradation products or abortive translations) are retained on GST-Corto and GST-C1/324. Right : Schematic representation of Corto (blue : chromodomain).

Next, we asked whether DSP1 and Corto interact directly. First, DSP1 was submitted to far-western analysis using full-length radiolabeled Corto as a probe. The 386 amino acid DSP1 protein exhibits two polyglutamine series located in the 1–155 region, two HMG-boxes (HMG-A box: amino acids 171 to 246; HMG-B box: amino acids 258 to 336) and an acidic tail. As shown in Figure 4A, Corto was retained on full-length DSP1. In order to identify the domain(s) of interaction in the DSP1 protein, we used several truncated forms of DSP1. Neither deletion of the NH_2_-terminal polyglutamine regions nor that of the COOH-terminal acidic tail impaired Corto binding (see B22, C8, D16, E5, F33, J11, M2). In contrast, Corto did not bind to G81, L5 or N4. Our results allowed us to conclude that the minimal DSP1 sequence needed for Corto binding was an intact HMG-box preceded by twelve amino acids (F33 and J11 rather than G81 or L5).

The reverse experiment, *i. e. *migration and transfer of the GST-Corto fusion proteins incubated with radiolabeled DSP1, gave no conclusive results owing to the difficulty in renaturing Corto. We showed previously that the only noticeable domain of the 550 amino acid Corto protein is a chromodomain located in the NH_2_-terminal half (aminoacids 107 to 203) [36]. In order to characterize the Corto domains interacting with DSP1, we performed GST pull-down experiments using full-length or truncated forms of Corto fused to GST and radiolabeled full-length DSP1. The results are shown in Figure 4B. DSP1 was retained by the full-length Corto protein, corroborating the far-western results with DSP1. Moreover, DSP1 was retained by a GST fusion protein containing the NH_2_-terminal half of Corto (GST-C1/324), but was not detectably retained on the COOH-terminal half (GSTC325/550 and GST-C440/550). Interestingly, DSP1 was not retained on the Corto chromodomain (GST-C127/203).

Taken together, these results indicate that, *in vitro*, DSP1 and Corto directly interact through either the HMG-box A or the HMG-box B of DSP1 on the one hand, and the amino-terminal half of Corto on the other.

### *corto *and *dsp1 *interact genetically and participate in *Sex comb reduced (Scr) *regulation

To determine whether *corto *and *dsp1 *are involved in common functions, we analyzed their genetic interactions. *dsp1^1 ^*hemizygous males present several homeotic transformations, notably a partial transformation of T1 to T2 leg as shown by a reduced sex comb (average size 6 teeth, rather than the 10 to 11 in a wild-type strain), and a partial transformation of A6 to A5 as shown by the presence of bristles on the A6 sternite (25% of *dsp1^1 ^*males exhibit this phenotype) [37]. These phenotypes are related to a role of *dsp1 *in the regulation of *Scr *and *AbdB*, respectively. We observed that loss of *corto *strongly suppresses both homeotic phenotypes (Table 1). Together, these data suggest that *corto *is involved with *dsp1 *in *Hox *gene regulation. Notably, they suggest that both *corto *and *dsp1 *regulate *Scr *in the T1 leg imaginal disc.

Interestingly, *dsp1^1 ^*was previously shown to cause partial suppression of the gain-of-function allele *Scr^S ^*[37]. Better to understand the relationship between *corto *and *dsp1 *in the regulation of *Scr*, we analyzed genetic interactions between *corto *and *Scr*. We first used two loss-of-function alleles of *Scr*, the EMS-induced *Scr^1 ^*allele and *Df(3R)Scx4*, a deficiency of the distal end of the *Scr cis*-regulatory regions. Males heterozygous for either allele (*Scr*^1^*/*+ or *Df(3R)Sxc^4^/*+) exhibit a reduction in the size of the sex comb on the first leg with an average number of 6.5 teeth per comb. No modification of this kind was observed in *Scr^1^/corto*^- ^or *Df(3R)Sxc^4^/corto*^- ^males (data not shown). We next checked the interactions between *corto *and the gain-of-function allele *Scr Antp^Scx ^*(Table 2). In this allele, the insertion of a transposable element near the *Antp *P1 promoter disturbs *Scr *silencing in the T2 and T3 leg imaginal discs [3]. Indeed, 39% of *Antp^Scx^/+ *males exhibited an ectopic sex comb on the T2 leg, *i.e. *a transformation of T2 leg into T1 leg. This percentage increased to 83%, 95% or 100%, depending on the *corto *allele, in *Antp^Scx^/corto *males (Table 2). These results show that *corto*, like *PcG *genes, participates in the maintenance of *Scr *repression in the T2 and T3 leg imaginal discs and corroborates our previous results showing that *corto *interacts with some *PcG *genes for this ectopic sex comb phenotype [35,38].

### Corto and DSP1 bind the same region within the 10-kb XbaI fragment of the *Scr *regulatory sequences

**Figure 5:**
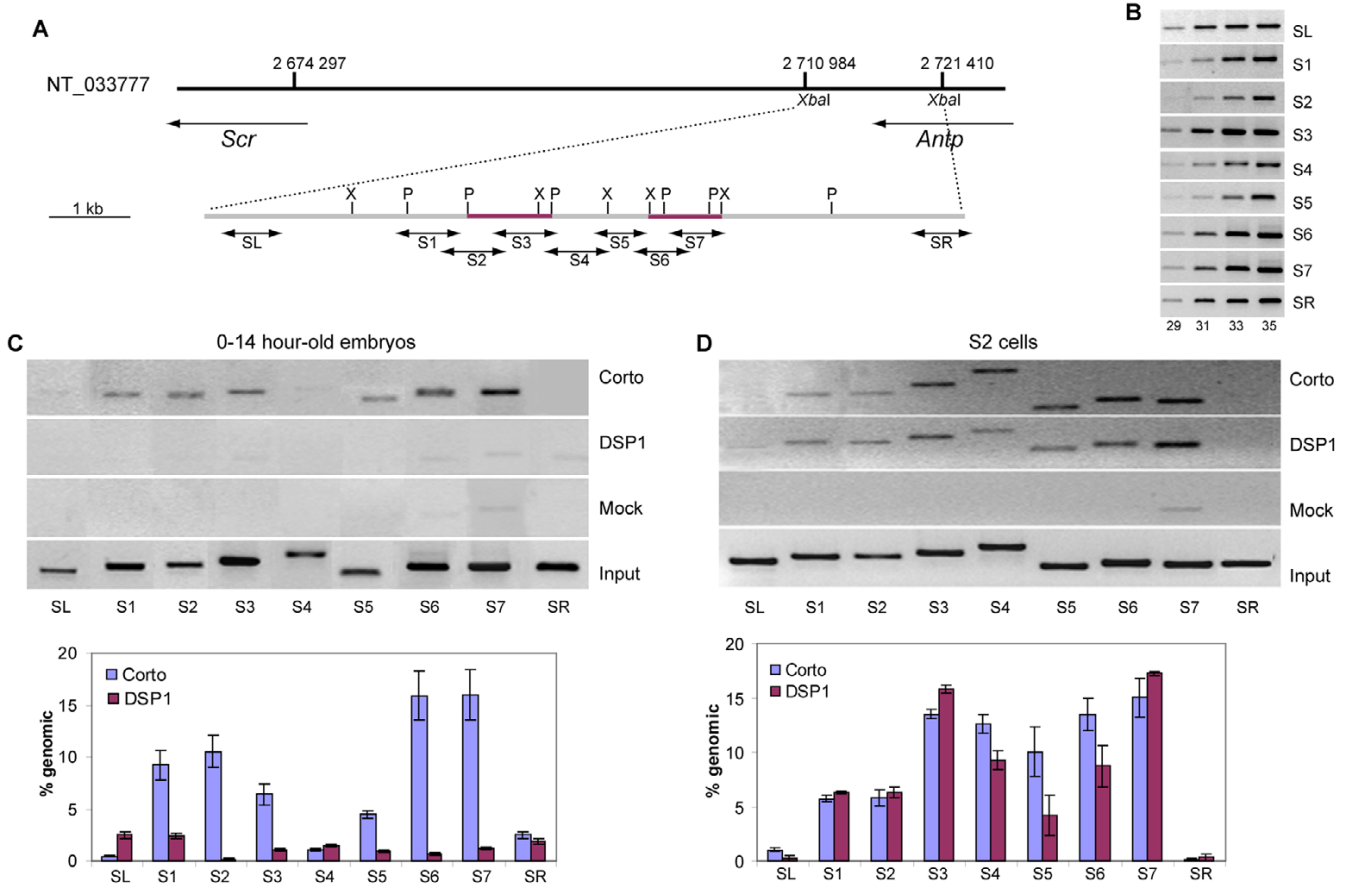
**Binding of Corto on the *Scr *10-kb *Xba*I fragment**. (A) The 10-kb *Xba*I *Scr *fragment is located about 37-kb upstream from the transcription start site. Numbers refer to NT_033777, *i.e. *the access number of the complete sequence of the *Drosophila melanogaster *3R chromosome. The 7 internal sequences (S1 to S7) and the two distal sequences (SL and SR) were amplified with specific primers as described in Methods. The *Pvu*II and *Xmn*I fragments, where DSP1 has been previously shown to bind, are in purple [41]. (B) To determine the linear range of amplification, PCR samples were taken at the 29^th^, 31^st^, 33^rd ^and 35^th ^PCR cycles. (C-D) Chromatin immunoprecipitation analysis of 0–14 hour-old embryos (C) or S2 cells (D) using rabbit anti-Corto, rabbit anti-DSP1 or rabbit serum as a control (mock). The 31^st ^cycle samples were loaded on a 1% agarose gel. The PCR products were quantified using Image J software and expressed as a percentage of the total input DNA. The diagrams represent the mean of 3 independent experiments.

To analyze the roles of Corto and DSP1 in *Scr *regulation further, we addressed their binding to *Scr cis*-regulatory sequences by chromatin immunoprecipitation experiments (XChIP). In S2 cells, XChIP followed by Southern analysis has previously shown that DSP1 binds two 1-kb sequences in the central part of a 10-kb *Xba*I fragment located about 37-kb upstream from the *Scr *transcription start [41]. Formaldehyde cross-linked chromatin from 0–14 hour-old embryos or S2 cells was immunoprecipitated with either DSP1 antibodies, Corto antibodies or rabbit serum as a control. Binding of Corto or DSP1 was analyzed by PCR amplification of 7 sequences (S1 to S7) covering the central region of the 10-kb *Xba*I fragment and two sequences (SL and SR) located on each side of this fragment. The results are shown in Figure 5. No binding of Corto or DSP1 was observed on the SL and SR fragments. DSP1 did not bind fragments S1 to S7 in embryos but bound them in S2 cells, corroborating previous results [41]. Maximum enrichment was observed for S3 and S7, which overlap the two 1-kb sequences where DSP1 binding was previously observed [41]. Corto binding was observed in both embryos and S2 cells. In embryos, we observed two main peaks of binding (S1–S2 and S6–S7). Interestingly, in S2 cells, Corto covered the entire S1 to S7 region. Therefore, the enlargement of the Corto binding domain was correlated with the presence of DSP1 on the same fragment.

## Discussion

We report in the present work that the two *ETPs corto *and *dsp1 *interact genetically and that the proteins they encode (i) directly interact *in vitro*, (ii) co-immunoprecipitate in embryos and (iii) co-localize on 91 sites in salivary gland polytene chromosomes. These results suggest that the proteins are simultaneously involved in the regulation of several target genes. DSP1 can bind Corto through one of the two HMG-boxes that also mediate DNA binding. It has been suggested that during nucleoprotein complex formation, the HMG-box B of HMGB bends DNA whereas the HMG-box A mediates interaction with transcription factors, thus promoting their contact with targets [22]. DSP1 seems to follow that scheme to enhance the binding of transcription factors as Dorsal or Bicoid to DNA [42,43]. What therefore could be the role of the DSP1-Corto interaction in the regulation of common targets? First, DSP1 could bring Corto to the chromatin, where it could further interact with other partners. These partners could be PcG factors or GAF, which have previously been shown to interact with Corto [36]. Nevertheless, this hypothesis is unlikely since we observed no modification of Corto binding to polytene chromosomes in the *dsp1^1 ^*strain. Second, DSP1 could inhibit the interaction between Corto and PcG factors or GAF, thus preventing the silencing of targets that bind both proteins. Third, Corto could modify the DNA bending ability of DSP1 and thus modulate its interaction with other factors, for example TrxG complexes. Indeed, the *dsp1 *gene was previously shown to interact with the *TrxG *genes *trx *and *brm *[37] Our results do not allow us to discriminate between these last two, non-exclusive possibilities.

We also report that the *Hox *gene *Scr *is a common target of Corto and DSP1. Both proteins bind a 10-kb *Xba*I fragment located 37-kb upstream of the *Scr *transcription start. Genetic studies have shown that this fragment is required for *Scr *function in the embryo and in the imaginal disc [44-46]. In embryos, it restricts the expression of a *Scr-lacZ *fusion gene to the labial and prothoracic segments [44], whereas in larvae it is required for *Scr *expression in the first leg imaginal disc and for *Scr *silencing in the second and third leg imaginal discs [45]. Interestingly, the function of the 10-kb *Xba*I fragment is sensitive to a subset of *PcG *and *TrxG *mutations [47] and has been genetically characterized as an upstream maintenance element of *Scr *[46]. At the end of embryogenesis, the *Scr *expression domain is restricted to the labial and prothoracic segments. In consequence, the mean state of this ME in the whole embryo would be silenced. We can thus assume that the global situation in embryos mimics that of the T2 and T3 leg imaginal discs. Conversely, since *Scr *is expressed in S2 cells (data not shown), we propose that the situation in S2 cells rather mimics that of T1 leg imaginal disc cells. Hence, Corto, which is present on the *Scr *ME whether active (S2 cells) or silenced (embryos), could be present on this ME in all three leg imaginal discs. On the other hand, DSP1, which is present on the ME in S2 cells but not in embryos, could bind the ME only in cells where this element is active, hence in T1 leg imaginal disc cells. We thus propose that both Corto and DSP1 proteins localize on this *Scr *ME in the first leg imaginal disc.

Some *trxG *mutants as well as the *dsp1 *null mutant exhibit a reduced sex comb and our previous work has shown that *dsp1 *interacts with certain *trxG *genes and regulates *Scr *expression in T1 discs [2,37]. HMGB, the vertebrate homologue of DSP1, has been reported to activate and stabilize the TFIID-TFIIA-promoter complex *in vitro *[48] and some TrxG factors have been shown to interact with the RNA polymerase II complex, thus facilitating transcriptional elongation [49,50]. This leads us to propose that in the T1 leg imaginal disc, DSP1 facilitates the interaction between a TrxG complex and the transcription machinery, thus maintaining *Scr *activation. Moreover, Corto has been shown to interact with PcG complexes [36]. The binding of Corto to DSP1 could then impede the interaction between Corto and PcG complexes, thus limiting their recruitment. Therefore, the interaction between the two proteins on the ME would lead to a level of *Scr *transcription compatible with T1 identity. Conversely, in the T2 and T3 leg imaginal discs, since DSP1 does not bind the ME, Corto would be able to interact with PcG complexes, thus enhancing the silencing of *Scr*.

## Conclusion

In summary, we have shown that the two ETPs Corto and DSP1 directly interact and are simultaneously found on a *Scr *ME when active, whereas Corto alone is found on the same ME when inactive. Our data suggest that different combinations of ETP favor the recruitment of either PcG or TrxG complexes, participating in the maintenance of the silenced or active state of ME.

## Methods

### Drosophila strains and genetics

Flies were raised on standard medium at 25°C except for the *dsp1 *strain, which was maintained at 22°C. All mutations and chromosome aberrations used are described in Flybase [51]. *Oregon-R *or *w^1118 ^*were used as wild-type reference strains. *dsp1^1 ^*is a null allele of *dsp1*, which is maintained as a homozygous strain [37]. *corto^420^*, *corto^07128 ^*and *corto^L1 ^*are loss-of-function alleles: a deficiency, a P-element insertion and an uncharacterized mutation obtained by EMS mutagenesis, respectively. These alleles were balanced over *TM3*.

### Localization of proteins on polytene chromosomes

Co-immunostaining of *Oregon-R *or *dsp1^1 ^*polytene chromosomes was performed as previously described using rabbit affinity purified anti-DSP1 (1:150) and rat anti-Corto (1:40) as primary antibodies [36]. Secondary antibodies (Alexa Fluor^® ^594 goat anti-rabbit IgG and Alexa Fluor^® ^488 goat anti-rat IgG, Molecular Probes) were used at a 1:1000 dilution. To determine the precise localization of Corto and DSP1, immunostainings were performed separately on squashes of *Oregon-R *chromosomes using rabbit affinity-purified anti-DSP1 (1:150) or rabbit anti-Corto (1:40) as described [40].

### Co-immunoprecipitation assays

Embryos (0–14 hour-old *w^1118^*, 2 g) were crushed in 4 ml of 50 mM Tris pH 7.5, 150 mM NaCl, 1% NP40, 0.1% SDS, 1 mM PMSF supplemented with protease inhibitors (Roche). After sonication and high-speed centrifugation, the extracts were pre-cleared with protein A-agarose beads and incubated overnight at 4°C with 10 μg of anti-DSP1 affinity-purified antibodies or 10 μl of rabbit serum. Following extensive washing, the beads were resuspended in Laemmli buffer and analyzed by SDS-PAGE and western blotting.

### Far-western and GST pull-down assays

Most of the vectors expressing full-length or truncated forms of MBP-DSP1 or GST-Corto fusion proteins have been described previously [36,37]. pGEX-C1/324 was obtained by digestion of pEG-Corto with *Eco*RI and *Pvu*II and sub-cloning the resulting 1-kb fragment into pGEX4T-1. pGEX-C325/550 was obtained by PCR amplification of a 0.7 kb DNA fragment from pBS-Corto using oligonucleotides 5'-CCG GAA TTC CGG GCT GCG GCC CAG GCC TCG ATA GCC-3' and 5'-CCG CTC GAG CGG CAC GTT GTA GCA GGA GAT CTG CGG-3', digestion with *Eco*RI and *Xho*I, and sub-cloning into pGEX4T-1. *In vitro *synthesis of radiolabeled proteins was performed using the TNT^® ^coupled reticulocyte lysate system (Promega) and ^35^S methionine. Far-western and GST pull-down assays were performed as previously described [36,37].

### Immunoprecipitation of crosslinked chromatin (XChIP) and semi-quantitative PCR analysis

Formaldehyde cross-linking of *Drosophila *S2 cell or 0–14 hour-old *w^1118 ^*embryo chromatin and chromatin immunoprecipitation were performed as described [52]. Immunoprecipitation of purified chromatin was performed either with rabbit Corto antibodies (1:20), affinity-purified DSP1 antibodies (5 μg) or rabbit serum (1:20) in 250 μl final volumes. One percent of the co-immunoprecipitated DNA was used for the PCR reactions. Nine primer pairs amplifying 500–700 bp fragments in the *cis*-regulatory sequences of *Scr *(NCBI accession number: NT_033777) were designed: SL forward 5'-AAA TCG GAC TGC TGC AAT TGA AGG-3', reverse 5'-AAT CCA AAA TGT TAC CCG GTG GCC-3', S1 forward 5'-GCA TCA AAA ACG AGT TAA GG-3', reverse 5'-ATA AAT CTT AGC TGC CTG CG-3', S2 forward 5'-CGC AGG CAG CTA AGA TTT ATG G-3', reverse 5'-CCG TTT GGG ATA AAC TTG GG-3', S3 forward 5'-GTT CCC AAG TTT ATC CCA AAC G-3', reverse 5'-GCT GAG GAG AAA GCT TCT GG-3', S4 forward 5'-CCA GAA GCT TTC TCC TCA GC-3', reverse 5'-CGG TAG CTG ATT TCG GAA AG-3', S5 forward 5'-CTT TCC GAA ATC AGC TAC CG-3', reverse 5'-TAT TTC CTC TCG AAC CCA CG-3', S6 forward 5'-GAG AGG AAA TAT GCA CTG GC-3', reverse 5'-GAA CGG ATC CAA ACC AAA CC-3', S7 forward 5'-AAA AGC TCT GAA AGT GTT AAG TGG C-3', reverse 5'-AAT GGC CCG AAT GAA GAA GC-3', and SR forward 5'-TCT ATC GTT CGG TTC AAT GGC TCC-3', reverse 5'-TTG GAA AAT GCC CAG CAG ACA TCC-3'. The PCR schemes were as follows: 94°C for 3 min; 94°C for 1 min, 57°C (S3, S6), 58°C (S4), 59°C (S1), 60°C (SL, S2, S5, S7, SR) for one min, 35 times, 72°C for one min; 72°C for 10 min. PCR samples were taken at the 29^th^, 31^st^, 33^rd ^and 35^th ^cycles to ensure that quantifications were performed in the linear range. PCR gels were photographed with a Biorad Geldoc system and quantified using ImageJ software (NIH). The results were normalized against the mock immunoprecipitation and presented as percentages of input DNA.

## Authors' contributions

JS performed the fluorescent immunodetection of DSP1 and Corto on polytene chromosomes and chromatin immunoprecipitation assays together with MB. MD and DL performed the genetic interactions between *corto *and *dsp1*. EMV performed the GST pull-down assays. AD performed the far-western experiments. LB and IZ performed the immunodetection of DSP1 and Corto on polytene chromosomes and determined the Corto and DSP1 binding sites. FP performed the genetic interaction between *corto *and *Scr*. The manuscript was written by FP; all the co-authors reviewed and approved the final manuscript.

## Figures and Tables

**Table 1:** Genetic interactions between *dsp1 *and *corto*

Genotype	Legs observed	average sex comb teeth on the T1 leg	Males observed	A6 to A5 (%)
+/*Y*	52	11.1	36	0
*dsp1^1^*/*Y *; +/+	40	6.0	46	25
*+*/*Y*; *corto^420^*/*+*	61	10.5	37	0
*+*/*Y*; *corto^07128^*/*+*	48	11.2	34	0
*dsp1^1^*/*Y*; *corto^420^*/+	113	9.0	68	0
*dsp1^1^*/*Y*; *corto^07128^*/+	31	9.0	69	3

Homozygous *dsp1*^*1 *^females were crossed with either *corto^420^*/*TM3 *or *corto^07128^*/*TM3 *males, or with *Oregon-R *males as controls. The resulting male progeny was scored for two homeotic transformations previously observed in *dsp1^1 ^*mutants: (i) transformation of T1 leg into T2 leg monitored by the size of the sex comb, (ii) transformation of segment A6 into segment A5 as revealed by the presence of bristles on A6 sternites.

**Table 2:** Genetic interactions between *corto *and *Scr*

Genotype	Males observed	T2 to T1 (%)
*Antp^Scx^*/+	163	38.6
*Antp^Scx^/corto^420^*	177	83.0
*Antp^Scx^/corto^07128^*	179	94.9
*Antp^Scx^/corto^L1^*	173	100.0

*Antp^Scx^/TM3 *females were crossed with *corto^420^/TM3, corto^07128^/TM3 *or *corto^L1^/TM3 *males, or *w^1118 ^*males as controls. Reciprocal crosses were also performed. As similar results were obtained, they were pooled. The resulting male progeny was scored for transformation of T2 into T1 leg as revealed by the presence of ectopic sex comb teeth.
